# A Fatal Case of Puumala Virus Infection with Pulmonary and Renal Syndrome in Moscow Region, Russia

**DOI:** 10.3390/pathogens15030321

**Published:** 2026-03-17

**Authors:** Ekaterina Blinova, Tamara Dzagurova, Galina Gopatsa, Natalya Pshenichnaya, Evgeny Morozkin, Vasiliy Akimkin

**Affiliations:** 1 Federal Budget Institute of Science «Central Research Institute of Epidemiology», Federal Service for Surveillance on Consumer Rights Protection and Human Wellbeing, 111123 Moscow, Russiavgakimkin@yandex.ru (V.A.); 2 Chumakov Federal Scientific Center for Research and Development of Immune-and-Biological Products, Russian Academy of Sciences (Institute of Poliomyelitis), 117218 Moscow, Russia; 3 State Budgetary Healthcare Institution of Moscow “Infectious Clinical Hospital No. 1 of the Department of Healthcare of Moscow”, 125310 Moscow, Russia; 4 Russian Medical Academy of Continuous Professional Education, Ministry of Health of the Russian Federation, 125993 Moscow, Russia

**Keywords:** hemorrhagic fever with renal syndrome, Puumala virus, reassortment

## Abstract

Hemorrhagic fever with renal syndrome (HFRS) is the most common zoonotic disease in Russia, with about a 98% abundance of Puumala virus in all HFRS cases. We report clinical manifestations and genomic characteristics of the Puumala virus strain that caused an unconventional course of HFRS with sudden death. The patient was admitted to the hospital on the third day from fever onset with hyperthermia, cough, shortness of breath, and severe weakness, and died 28 h after hospitalization despite intensive care. Further analyses of autopsy samples led to Puumala virus detection. The viral genome was sequenced, followed by phylogenetic and similarity plot analyses. The genomic sequences of three viral segments were identified as endemic for the Moscow region strain. Phylogenetic and similarity plot analysis revealed the reassortant origin of the strain via M segment exchange. This finding increases the explored molecular diversity of Puumala virus in the Central Federal District and underscores the need for heightened awareness of HFRS manifestations that deviate from regular clinical presentation.

## 1. Introduction

Several Orthohantaviruses (family Hantaviridae) are considered dangerous to humans because of their clinical significance and cause hemorrhagic fever with renal syndrome (HFRS), which is the most common zoonosis in Russia [1]. Six hantaviruses cause HFRS in Russia: Puumala, Hantaan, Amur, Seoul, Kurkino, and Sochi viruses. The vast majority of HFRS cases in Russia, more than 97% of all cases, are caused by Puumala virus (PUUV) [2]. The mild form of HFRS PUUV is known in Europe as nephropathia epidemica (NE) [3]. In Russia, Puumala virus causes HFRS of varying clinical manifestations: about 25% cases are mild, 25% severe, and 50% are of moderate severity [4]. Diapedesis and other hemorrhagic manifestations, hemodynamic disorders, and kidney damage are characteristic of HFRS PUUV. In severe cases, the widespread damage to small vessels leads to multiple organ failure [4].

The current diversity of the PUUV is subdivided into eight lineages: Central European (CE), Alpe-Adrian (ALAD), Danish (DAN), South Scandinavian (S-SCAN), North Scandinavian (N-SCAN), Finnish (FIN), Russian (RUS), and Latvian (LAT) [5]. Two of them are known in the territory of the Russian Federation: RUS (Volga-RUS and W-RUS sublineages) and FIN (West-FIN and East-FIN sublineages) [6]. In Russia, the genetic diversity of the Puumala virus has been well studied in the most intense HFRS-focused region in Volga Federal District, where the Volga-RUS sublineage circulates.

Although Central Federal District accounts for nearly 13% of the total HFRS cases in Russia [7], the PUUV genome variants had not been investigated on this territory until recently. The latest studies have shown that the genetic variants of the Puumala virus from the Central Federal District form a separate sublineage—W-RUS [6]. Reassortment events were found between [6] and within Volga-RUS [8,9,10,11] and W-RUS [6] sublineages, as well as in other PUUV lineages [12]. Recombinant strains emerge continuously in nature during circulation in host animal populations [13,14,15]. Therefore, the study of PUUV genetic diversity in the Central Federal District of Russia is still relevant, since only a few variants of the W-RUS sublineage genome are known.

In this study, we aimed to investigate a fatal case of HFRS caused by a PUUV strain from the Moscow region that presented with this unusual disease manifestation.

## 2. Materials and Methods

Autopsy fragments of the patient’s liver, spleen, kidney, and lung were received on the 3rd day after death (storage and transportation at +4 °C for 57 h). They were processed using a homogenizer with sterile saline solution to obtain 10% organ suspensions. Total RNA was extracted using a RIBO-prep reagent kit (Central Research Institute of Epidemiology of Rospotrebnadzor, Moscow) in accordance with the manufacturer’s instructions and analyzed by S segment-targeted real-time RT-PCR [6].

For amplification and sequencing, the RNA was repeatedly isolated from the liver suspension by phenol-chloroform extraction. The viral RNA was reverse-transcribed, and cDNA was amplified using 24 pairs of primers as previously described [6]. The PCR products were sequenced by the Sanger method using a BigDye Terminator v1.1 Cycle Sequencing kit (Thermo Fisher Scientific, Austin, TX, USA) on an Applied Biosystems 3500xL Genetic Analyzer (Applied Biosystems, Foster City, CA, USA).

DNASTAR Lasergene SeqMan version 7.0.0 software was used for the processing of sequencing data. For phylogenetic analysis, the sequences were aligned using the Muscle algorithm in the MEGA X program version 10.2.6 [16]. The phylogenetic trees were constructed using the maximum likelihood method with the general time reversible (G + I) model with a bootstrap value of 1000 using the MEGA X program. The similarity plot were constructed using Simplot 3.5.1 [17].

## 3. Results

The 52-year-old resident of a private home in a village in the Leninsky district of the Moscow region experienced pain in the right lumbar region radiating to the right groin area and right hip joint. These symptoms disappeared after anti-inflammatory and analgesic injections.

Four days later, the patient developed a fever (39–40 °C), cough, profuse sweating, weakness, shortness of breath, thirst, and minor muscle pain. Due to increased symptoms, he was hospitalized on the third day of fever on an emergency basis. As rapid tests for influenza A/B and SARS-CoV-2 antigens showed negative results, ICD code J18.9 (unspecified) pneumonia was diagnosed.

On admission, the patient was in a state of lethargy with hyperemic skin, swelling of the eyelids, hypotension 95/60 mmHg, thirst, decreased diuresis, and loose stool. No rashes or other hemorrhagic manifestations on the skin or mucous membranes were observed. A CT scan revealed focal consolidations in the upper lobes of both lungs. A clinical blood test upon admission showed: platelets—58.0 (N 150–400) × 10^9^/L, leukocytes—12.63 (N 4–9) × 10^9^/L (neutrophilic shift), creatinine 204 (N 72–127) μmol/L, alanine aminotransferases—60 (N 0–35) IU, and aspartate aminotransferases—85 (N 0–35) IU. Urine analysis showed: protein—5 g/L, leukocytes—18, and bacteria in significant quantities per high-power field.

The patient had the following concomitant diseases: ICD code I25.5 ischemic cardiomyopathy and old myocardial infarction (OMI), with the precise date unknown. Chronic diseases: arterial hypertension.

Epidemiological history: Trip to Western Siberia (Khanty-Mansiysk Autonomous Okrug) about a month before the disease onset. The presence of rodents in the place of residence. No animal or insect bites. Medical history: acute respiratory viral infection, mumps, rubella, chickenpox.

According to the ultrasound results, 20 h after hospitalization, free fluid was detected in the pleural cavities on both sides at the 6–7 intercostal level. At 26 h after admission, according to a clinical blood test, the results were: platelets—24.0 × 10^9^/L, leukocytes—29.25 × 10^9^/L, and creatinine 440.0 µmol/L.

Supportive (electrolytes, epinephrine) and antibacterial (ceftriaxone, cefoperazone + sulbactam) treatment was started. Due to deterioration of the condition, mechanical ventilation and hemodialysis were started. Despite all efforts, the patient died 28 h after hospitalization.

Main diagnosis: ICD code A98.5 hemorrhagic fever with renal syndrome. Bacterial infection, unspecified, severe. Cerebral and pulmonary edema.

**Figure 1:**
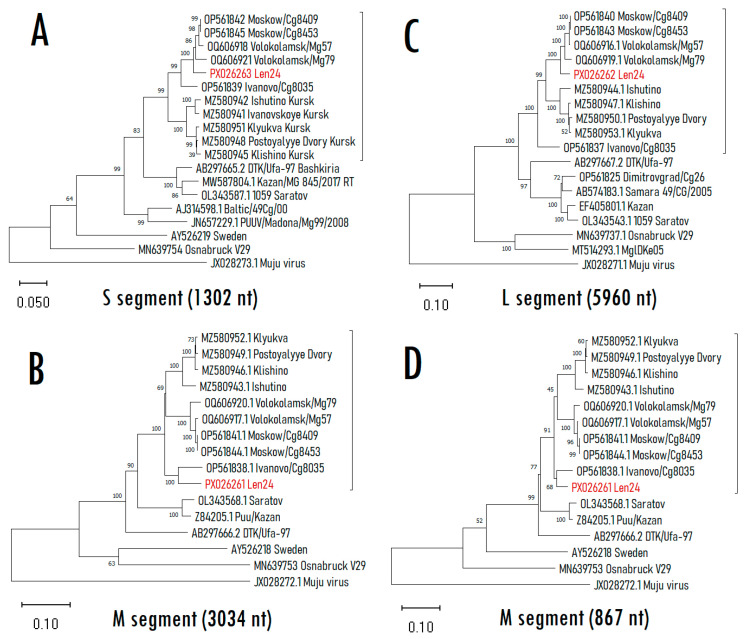
Phylogenetic trees of small (S), medium (M), and large (L) segments. (**A**) S segment based on complete open reading frame (ORF) of 1302 nt; (**B**) M segment based on partial ORF 3034 nt; (**C**) L segment based on partial ORF 5960 nt, (**D**) M segment based on partial ORF 1–867 nt. Values in parentheses represent the W-RUS sublineage. The phylogenetic trees were constructed using the maximum likelihood method using the general time reversible (G + I) model with a bootstrap value of 1000 using the MEGA X program.

Autopsy samples from liver, spleen, kidney, and lung were analyzed by RT-PCR. Puumala virus RNA was detected in all examined organs, with cycle threshold values ranging from 21 to 23. We obtained sequences of L, M, and S segments with lengths of 6409, 3628, and 1514 pairs of nucleotides, respectively (PX026261–PX026263). Phylogenetic analysis of each of the segments showed that the sequences obtained belonged to the recently discovered sublineage W-RUS, which is endemic to the Moscow region (Figure 1).

**Figure 2:**
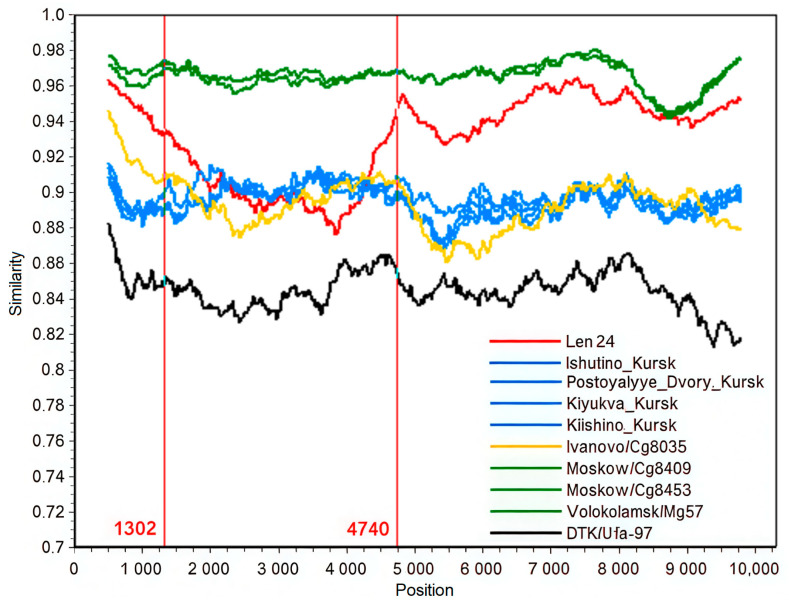
Similarity plot against query sequence (Volokolamsk/Mg79). Concatenated sequences of full S segment ORF (1302 nt, 1–1302 position), full M segment ORF (3447 nt, 1303–4749 position), and partial L segment ORF (1–6409 nt, 4750–11,158 position) were analyzed. Window 1000 nt, step 20 nt.

The sequences of the L and S segments of the Len24 isolate exhibited close phylogenetic proximity to other sequences originating from the Moscow region (Volokolamsk/Mg79 Volokolamsk/Mg57, Moskow/Cg8409, Moskow/Cg8453). At the same time, the sequence of the M segment was grouped with the Ivanovo/Cg8035 isolate. These data suggest that the obtained sequence may have originated as a result of reassortment. To confirm this suggestion, we compared similarity among several strains (Figure 2).

The graph shows a low similarity between new sequence Len24 and strains from the Moscow region (the query and green group) in the M segment in comparison with the S and L segments (Figure 2). The intersections of Len24 and of sequences from Kursk (blue group) graphs between 800 and 1000 nt of the M segment ORF (depending on window size) also indicate a possible recombination event between their ancestors. However, the phylogenetic tree based on the first 857 nucleotides of the M segment (Figure 1D) did not confirm an exchange of RNA regions within the segment. The S and L segments of the new strain apparently originate from a common ancestor of Moscow region isolates (Moskow/Cg8409, Moskow/Cg8453, Volokolamsk/Mg57, Volokolamsk/Mg79) while the M segment originates from other genome variant via reassortment.

## 4. Discussion

The absence of any manifestations of hemorrhagic syndrome was atypical for HFRS, as well as cough and shortness of breath at the initial stage of the disease [4,18,19], which were previously described in rare cases of Puumala infection manifesting as hantavirus pulmonary syndrome (HPS) [20,21,22]. At the same time, acute onset of the disease with febrile temperature and hemodynamic disturbances in the form of hypotension, decreased diuresis, and pain in the lumbar region are typical symptoms of HFRS, alongside high creatinine level and acute decreases in platelet and leukocyte counts on blood analysis [18,19,23]. Lung involvement is also often observed in HFRS cases of Puumala infection [24,25,26,27].

Viral RNA was detected in all the organs studied. The results of the ultrasound examination revealed echo signs of enlargement and diffuse changes in the liver and in the pancreas, which may be a consequence of a viral infection, and pleural effusion. The results of real-time RT-PCR of autopsy samples indicated that viral RNA quantity in the studied samples differs no more than fourfold. This contrasts with results of cases with multiple organ failure, where a significant difference in the content of RNA in organs was detected [28]. The tropism of the virus to various organs, as well as the course and outcome of the disease, may depend on various factors, such as the peculiar properties of a strain or immune response.

There was evidence about the patient’s visit to Western Siberia (Khanty-Mansiysk Autonomous Okrug) about a month before the onset of the disease, which matches the 7–46 days of incubation period of HFRS [4]. However, the East-FIN sublineage of PUUV could be endemic for that region [6]. The affiliation of the obtained sequences with the W-RUS sublineage indicates a more plausible infection in the Moscow region at the patient’s place of residence. The presence of mice in the private home where the patient lived testifies to this assumption.

Interestingly, the S and L segments of the obtained sequences clustered with other sequences originating from the Moscow region (Volokolamsk/Mg79 Volokolamsk/Mg57, Moskow/Cg8409, Moskow/Cg8453) in the same configuration. However, the M segment was grouped with the strain from Ivanovo region (Ivanovo/Cg8035) into an external group in relation to isolates from the Moscow and Kursk regions. At the same time, apparently, the strain Ivanovo/Cg8035 appears to be reassortant in itself. Indeed, the Ivanovo/Cg8035 S segment sequence is related to the clade from the Moscow region (Figure 1A), while its M and L segments are an outgroup of Moscow and Kursk clades (Figure 1B,C).

The positions of the new sequences within the W-RUS clade on all three trees had high nodal support (more than 99), which indicates the reliability of the conclusions about the reassortment. The similarity plot (Figure 2) also supports this result. This phylogenetic contradiction was discovered using a rather large window size (1000 nucleotides), which confirms the validity of the finding.

Indeed, it was found that strains circulating in geographically close populations can be shuffled and generate reassortant genotypes [10,13,15]. However, the parental genotypes of the described reassortant strain belong to rather remote territories of Ivanovo and Moscow regions (more than 300 km). Virus strain dissemination could be associated with the transfer of rodent vectors via active transport of grain crops.

Despite numerous reports of intraspecific reassortment of the Puumala virus, the changes in the pathogenic properties of the resulting strains in comparison with the parent ones remains unclear. Further study of the genetic diversity of the Puumala virus in the Central Federal District may shed light on this issue and on the evolutionary processes in the W-RUS sublineage.

Several HFRS reports have already described non-cardiogenic HPS-like lung involvement prior to any kidney involvement [25,26,27,28]. Our message complements this information with new evidence of a significant clinical overlap between HPS and HFRS.

## 5. Conclusions

A patient with fatal HFRS-PUUV was infected by a reassortant strain of PUUV belonging to the W-RUS sublineage. The clinical presentation was marked by the absence of hemorrhagic syndrome and the presence of pulmonary involvement resembling HPS, which complicated the initial diagnosis. Phylogenetic analysis revealed that the strain originated from a reassortment event, acquiring its M segment from a variant circulating in the Ivanovo region, while its S and L segments originated from local Moscow strains. This finding highlights the potential for reassortment to generate strains with unpredictable pathogenic properties. Further surveillance and genetic characterization of circulating hantaviruses are essential to better understand their evolution, epidemiology, and impact on clinical outcomes.

## Data Availability

The data presented in this study are available on NCBI (National Center for Biotechnology Information) GenBank^®^ at https://www.ncbi.nlm.nih.gov/nucleotide (accessed on 5 January 2026) (reference PX026261–PX026263). The raw data supporting the conclusions of this article will be made available by the authors on request.

## Abbreviations

The following abbreviations are used in this manuscript.

HFRS	hemorrhagic fever with renal syndrome
HPS	hantavirus pulmonary syndrome
ORF	open reading frame
OMI	old myocardial infarction

## References

[1] Garanina S.B., Platonov A.E., Zhuravlev V.I., Murashkina A.N., Yakimenko V.V., Korneev A.G., Shipulin G.A. (2009). Genetic diversity and geographic distribution of hantaviruses in Russia. Zoonoses Public Health.

[2] Tkachenko E., Kurashova S., Balkina A., Ivanov A., Egorova M., Leonovich O., Popova Y., Teodorovich R., Belyakova A., Tkachenko P. (2023). Cases of Hemorrhagic Fever with Renal Syndrome in Russia during 2000–2022. Viruses.

[3] Castel G., Couteaudier M., Sauvage F., Pons J.-B., Murri S., Plyusnina A., Pontier D., Cosson J.-F., Plyusnin A., Marianneau P. (2015). Complete Genome and Phylogeny of *Puumala Hantavirus* Isolates Circulating in France. Viruses.

[4] Morozov V.G., Ishmukhametov A.A., Dzagurova T.K., Tkachenko E.A. (2017). Clinical manifestations of hemorrhagic fever with renal syndrome in Russia. Med. Counc..

[5] Razzauti M., Plyusnina A., Niemimaa J., Henttonen H., Plyusnin A. (2012). Co-circulation of two *Puumala hantavirus* lineages in Latvia: A russian lineage described previously and a novel Latvian lineage. J. Med. Virol..

[6] Blinova E., Deviatkin A., Makenov M., Popova Y., Dzagurova T. (2023). Evolutionary Formation and Distribution of Puumala Virus Genome Variants, Russia. Emerg. Infect. Dis..

[7] Savitskaya T.A., Ivanova A.V., Isaeva GSh Reshetnikova I.D., Trifonov V.A., Ziatdinov V.B., Serova I.V., Safronov V.A. (2020). Assessment of epidemiological situation on hemorhhagic fever with renal syndrome around the world and in Russia, forecast for 2020. Probl. Espec. Danger. Infect..

[8] Davidyuk Y., Shamsutdinov A., Kabwe E., Ismagilova R., Martynova E., Belyaev A., Shuralev E., Trifonov V., Savitskaya T., Isaeva G. (2020). Prevalence of the *Puumala orthohantavirus* strains in the pre-kama area of the Republic of Tatarstan, Russia. Pathogens.

[9] Davidyuk Y.N., Kabwe E., Shamsutdinov A.F., Knyazeva A.V., Martynova E.V., Ismagilova R.K., Trifonov V.A., Savitskaya T.A., Isaeva G.S., Urbanowicz R.A. (2021). The Distribution of *Puumala orthohantavirus* Genome Variants Correlates with the Regional Landscapes in the Trans-Kama Area of the Republic of Tatarstan. Pathogens.

[10] Kabwe E., Shamsutdinov A.F., Suleimanova S., Martynova E.V., Ismagilova R.K., Shakirova V.G., Savitskaya T.A., Isaeva G.S., Rizvanov A.A., Khaiboullina S.F. (2023). Puumala Orthohantavirus Reassortant Genome Variants Likely Emerging in the Watershed Forests. Int. J. Mol. Sci..

[11] Kabwe E., Al Sheikh W., Shamsutdinov A.F., Ismagilova R.K., Martynova E.V., Ohlopkova O.V., Yurchenko Y.A., Savitskaya T.A., Isaeva G.S., Khaiboullina S.F. (2022). Analysis of *Puumala orthohantavirus* Genome Variants Identified in the Territories of Volga Federal District. Trop. Med. Infect. Dis..

[12] Klempa B. (2018). Reassortment events in the evolution of hantaviruses. Virus Genes.

[13] Razzauti M., Plyusnina A., Henttonen H., Plyusnin A. (2013). Microevolution of *Puumala hantavirus* during a Complete Population Cycle of Its Host, the Bank Vole (*Myodes glareolus*). PLoS ONE.

[14] Razzauti M., Plyusnina A., Sironen T., Henttonen H., Pyusnin A. (2009). Analysis of *Puumala hantavirus* in a bank vole population in northern Finland: Evidence for co-circulation of two genetic lineages and frequent reassortment between strains. J. Gen. Virol..

[15] Razzauti M., Plyusnina A., Henttonen H., Plyusnin A. (2008). Accumulation of point mutations and reassortment of genomic RNA segments are involved in the microevolution of *Puumala hantavirus* in a bank vole (*Myodes glareolus*) population. J. Gen. Virol..

[16] Kumar S., Stecher G., Li M., Knyaz C., Tamura K. (2018). MEGA X: Molecular Evolutionary Genetics Analysis across Computing Platforms. Mol. Biol. Evol..

[17] Lole K.S., Bollinger R.C., Paranjape R.S., Gadkari D., Kulkarni S.S., Novak N.G., Ingersoll R., Sheppard H.W., Ray S.C. (1999). Full-Length Human Immunodeficiency Virus Type 1 Genomes from Subtype C-Infected Seroconverters in India, with Evidence of Intersubtype Recombination. J. Virol..

[18] Bondarenko A.L., Abbasova S.V., Korobitsyn K.G. (2015). Hemorrhagic fever with renal syndrome in the Kirov region at the present stage. Epidemiol. Infect. Deseases.

[19] Suzdaltsev A.A., Morozov V.G., Lukaev R.R., Tkachenko E.A. (2014). Hemorrhagic fever with renal syndrome (puumala) in the natural focuses at the territory of central volga area: Dynamics of clinical and laboratory manifestations over the period of 1997–2012. Infect. Dis. News Opin. Educ..

[20] Santini M., Ljubić J., Šoštar N., Vilibić-Čavlek T., Bogdanić M., Zakotnik S., Avšič-Županc T., Korva M., Kurolt I.C., Radmanić L. (2023). Hantavirus Pulmonary Syndrome Caused by Puumala Orthohantavirus—A Case Report and Literature Review. Microorganisms.

[21] Sulleiro E., Aznar M.L., Serre-Delcor N., Salvador F., Sanchez-Montalvá A., Espasa M., Molina D., de Ory F., Sanchez-Seco M.P., Molina I. (2020). Hantavirus Pulmonary Syndrome in Traveler Returning from Nepal to Spain. Emerg. Infect. Dis..

[22] Rasmuson J., Andersson C., Norrman E., Haney M., Evander M., Ahlm C. (2011). Time to revise the paradigm of hantavirus syndromes? Hantavirus pulmonary syndrome caused by European hantavirus. Eur. J. Clin. Microbiol. Infect. Dis..

[23] Mustonen J., Outinen T., Laine O., Pörsti I., Vaheri A., Mäkelä S. (2017). Kidney disease in *Puumala hantavirus* infection. Infect. Dis..

[24] Rasmuson J., Lindqvist P., Sörensen K., Hedström M., Blomberg A., Ahlm C. (2013). Cardiopulmonary involvement in *Puumala hantavirus* infection. BMC Infect. Dis..

[25] Clement J., Maes P., Van Ranst M. (2014). Hemorrhagic Fever with Renal Syndrome in the New, and Hantavirus Pulmonary Syndrome in the old world: Paradi(se)gm lost or regained?. Virus Res..

[26] Gizzi M., Delaere B., Weynand B., Clement J., Maes P., Vergote V., Laenen L., Hjelle B., Verroken A., Dive A. (2013). Another case of “European hantavirus pulmonary syndrome” with severe lung, prior to kidney, involvement, and diagnosed by viral inclusions in lung macrophages. Eur. J. Clin. Microbiol. Infect. Dis..

[27] Launay D., Thomas Ch Fleury D., Roueff S., Line M.-L., Droz D., Vanhille P. (2003). Pulmonary-renal syndrome due to hemorrhagic fever with renal syndrome: An unusual manifestation of Puumala virus infection in France. Clin. Nephrol..

[28] Kruger D.H., Tkachenko E.A., Morozov V.G., Yunicheva Y.V., Pilikova O.M., Malkin G., Ishmukhametov A.A., Heinemann P., Witkowski P.T., Klempa B. (2015). Life-Threatening Sochi Virus Infections, Russia. Emerg. Infect. Dis..

